# Clinical impact of ^99m^Tc-MAA SPECT/CT-based personalized predictive dosimetry in selective internal radiotherapy: a real-life single-center experience in unresectable HCC patients

**DOI:** 10.1186/s41824-023-00171-8

**Published:** 2023-07-07

**Authors:** Ana-Maria Bucalau, Benoît Collette, Illario Tancredi, Michael Vouche, Martina Pezzullo, Jason Bouziotis, Rodrigo Moreno-Reyes, Nicola Trotta, Hugo Levillain, Jean Luc Van Laethem, Gontran Verset

**Affiliations:** 1 Department of Gastroenterology, Hepatopancreatology and Digestive Oncology, Hôpital Erasme/Bordet Institute-Hôpital Universitaire de Bruxelles, Université Libre de Bruxelles (ULB), Brussels, Belgium; 2 Department of Nuclear Medicine, Hôpital Erasme/Bordet Institute-Hôpital Universitaire de Bruxelles, Université Libre de Bruxelles (ULB), Brussels, Belgium; 3 Department of Radiology, Hôpital Erasme/Bordet Institute-Hôpital Universitaire de Bruxelles, Université Libre de Bruxelles (ULB), Brussels, Belgium; 4 Department of Biomedical Research, Hôpital Erasme/Bordet Institute-Hôpital Universitaire de Bruxelles, Université Libre de Bruxelles (ULB), Brussels, Belgium

**Keywords:** Selective Internal RadioTherapy, Transarterial radioembolization, Hepatocellular carcinoma, ^99m^Tc-MAA scintigraphy

## Abstract

**Background:**

Recent data demonstrated that personalized dosimetry-based selective internal radiotherapy (SIRT) is associated with better outcome for unresectable hepatocellular carcinoma (HCC).

**Aim:**

We aim to evaluate the contribution of personalized predictive dosimetry (performed with Simplicity^90^® software) in our population of HCC patients by comparing them to our historical cohort whose activity was determined by standard dosimetry.

**Methods:**

This is a retrospective, single-center study conducted between February 2016 and December 2020 that included patients with HCC who received SIRT after simulation based on either standard dosimetry (group A) or, as of December 2017, on personalized dosimetry (group B). Primary endpoints were best overall response (BOR) and objective response rate (ORR) evaluated by mRECIST at 3 months. Safety and toxicity profiles were evaluated at 1- and 3-months post-treatment. For group A we compared the activity to be administered determined a posteriori using Simplicit^90^Y® and the activity actually administered determined by the standard approach.

**Results:**

Between February 2016 and December 2020, 66 patients received 69 simulations leading to 40 treatments. The median follow-up time was equal for both groups, 21 months (range 3–55) in group A and 21 months (range 4–39) in group B. The per patient analysis revealed a significant benefit of personalized predictive dosimetry in terms of better overall response at 3 months (80% vs. 33.3%, *p* = 0.007) and at 6 months (77.8% vs. 22.2%, *p* = 0.06). This trend was found in the analysis by nodule with a response rate according to mRECIST of 87.5% for personalized dosimetry versus 68.4% for standard dosimetry at 3 months, *p* = 0.24. Only one grade 3 biological toxicity (hyperbilirubinemia) was noted in group A. The comparison between the administered activity and the recommended activity recalculated a posteriori using Simplicit^90^Y® showed that the vast majority of patients who progressed (83.33%) received less activity than that recommended by the personalized approach or an inadequate distribution of the administered activity.

**Conclusions:**

Our study aligns to recent literature and confirms that the use of personalized dosimetry allows a better selection of HCC patients who can benefit from SIRT, and consequently, improves the effectiveness of this treatment.

## Introduction

Selective Internal RadioTherapy (SIRT), known as TransArterial RadioEmbolization (TARE) in the context of liver disease, has a controversial role in the treatment of advanced hepatocellular carcinoma (HCC), as two phase III randomized controlled trials, comparing SIRT to Sorafenib, showed no difference in terms of overall survival (OS) (Vilgrain et al. 2017; Chow et al. 2018). Moreover, a phase II study concluded that the addition of SIRT to Sorafenib did not bring a benefit in terms of survival (Ricke et al. 2018). Only recently SIRT has regained a place in the Barcelona Clinic Liver Cancer (BCLC) staging system for solitary tumors less or equal to 8 cm, thanks to the LEGACY trial (Salem et al. 2021).

In all these studies the prescribed activity was based on the body surface area (BSA), a standard calculation method for the activity of ^90^Y to be administered that correlates with the patient’s liver volume, adjusted by the percentage of tumor involvement and the magnitude of the lung-shunt fraction (i.e. the fraction of injected microspheres lodged within the pre-capillary of the lungs) (Dezarn et al. 2011; Grosser et al. 2015). However, the BSA method may lead to underdosing or even overdosing due to its moderate correlation to the liver volume in patients with liver diseases (hepatomegaly or patients with liver resection prior to SIRT). Furthermore, the percentage of tumor involvement adds little value when adjusting the activity to administer.

In order to optimize the administered activity, the concept of a more personalized dosimetry has been advocated, making use of patient-specific parameters and a multi-compartmental modeling that estimates the dose to the tumor, based on the Medical Internal Radiation Dose (MIRD) model (Salem et al. 2019; Levillain et al. 2021; Weber et al. 2022).

The capacity to predict the distribution of ^90^Y-microspheres has been shown to be a factor of improvement of SIRT efficacy (Ho et al. 1997a; Ho et al. 1997b; Garin et al. 2016). The similar distribution of ^99m^Tc-macroaggregated albumin (^99m^Tc-MAA) and ^90^Y-microspheres allows dosimetry simulation using ^99m^Tc-MAA single-photon emission computed tomography (^99m^Tc-MAA SPECT/CT) co-registered with MRI, or contrast-enhanced CT (CE-CT).

In our institution glass microspheres (TheraSphere®, Boston Scientific) have been used since 2016, at first using a standard dosimetry-based simulation (i.e. total perfused volume dose-based dosimetry without any idea of the actual tumor dose), and, more recently, with a personalized dosimetry to prescribe a more accurate activity (i.e. calculated using Simplicit^90^Y®, a software able to do multi-compartmental MIRD dosimetry). According to recent publications, a mean absorbed dose of minimum 205 Gy delivered to the lesion, when treating patients with glass ^90^Y-microspheres, is required to achieve an optimal response (Garin et al. 2017; Gnesin et al. 2016). Furthermore, in 2021, the randomized, multicenter, open-label phase 2 Dosiphere-01 trial showed a significant improvement in objective response rate when using a personalized approach (Garin et al. 2021).

However, although randomized studies are the “gold standard” for the evaluation of safety and efficacy of any treatment, observational studies conducted in a real-world scenario bring precious evidence on the effectiveness of the treatment in clinical practice.

Therefore, we aim to determine if these recent results can be retrieved from a retrospective dataset in our tertiary hospital, by comparing the response rate of SIRT obtained using standard dosimetry-based simulation to that obtained with SIRT using personalized dosimetry-based simulation. In addition, for patients treated after standard dosimetry-based simulation, we compared the activity administered and the activity that would have been administered if personalized dosimetry had been applied.

## Material and methods

### Study design and patient selection

This is a retrospective study conducted in a tertiary health-center in Belgium, from February 2016 to December 2020, that enrolled consecutive HCC patients who underwent at least one work-up (simulation based on ^99m^Tc-MAA scintigraphy) for radioembolization and received or not the treatment by SIRT (by ^90^Y-loaded glass microspheres). Patients were divided in two consecutive groups: patients who underwent work-up with standard dosimetry-based simulation (group A) from February 2016 to November 2017 and patients who underwent work-up with personalized dosimetry-based simulation (group B) from December 2017 to last enrolment at the end of 2020.

The study was reviewed and approved by the local ethics committee (P2021/221). Due to the retrospective nature of the study, informed consent was not required.

Patients were referred from the multidisciplinary hepatology tumor board and met the following *inclusion* criteria: unresectable HCC not eligible for curative treatments (ablative treatments or surgical resection) with at least one measurable lesion (BCLC A, B and C), liver dominant or liver only disease; Child–Pugh score ≤ B7, Eastern Cooperative Oncology Group (ECOG) Performance Status score ≤ 1. *Exclusion criteria* included: lung absorbed dose > 30 Gy or uncorrectable extrahepatic deposition of the MAA activity identified at ^99m^Tc-MAA (whole body) scintigraphy, unmanageable intolerance to contrast medium and contraindication to hepatic angiography.

### Study procedures, activity calculation and dosimetry

The radioembolization procedure was performed over two different sessions: work-up session and treatment session, by the same two interventional radiologists with 5–10 years of experience (between 50–100 procedures per year), following the current standard of practice and according to the manufacturer’s instructions. The work-up evaluation started with an angiography in order to obtain a precise map of the patients’ abdominal vascular anatomy and coil embolization was performed if gastrointestinal branches arising from the hepatic arteries were found.

Patients in *group A* were treated with ^90^Y activity calculation based on a mono-compartmental dosimetry planning, using a volume based on a ^99m^Tc-MAA SPECT, after lung shunt fraction evaluation on a whole-body scan. For the standard predictive dosimetry of *group A*, a calculation sheet from the ^90^Y provider gave us the activity knowing the volume segmented from the ^99m^Tc-MAA SPECT data (with thresholding based on a maximum intensity percentage of 1%), the lung shunt fraction determined on the whole-body scan (with manual segmentation), and the desired dose to the targeted volume (total perfused volume, based on standard guidelines).

Patients in *group B* were treated after personalized predictive (multi-compartmental) dosimetry that was performed using Simplicit^90^Y® software. MRI or CE-CT was used for the segmentation of the liver, the tumor, and the non-tumoral liver (manually on MRI, automatically on CE-CT with corrections when needed). Then, ^99m^Tc-MAA SPECT/CT was co-registered and the perfused volume was determined (with thresholding based on a maximum intensity percentage of 1%) after lung shunt fraction evaluation on the whole-body scan.

Regarding the work-up, for both groups, ^99m^Tc-MAA were injected after diagnostic angiography in the hepatic artery (with activities between 150 and 200 MBq). Images were performed on a Philips BrightView XCT gamma-camera with a Low Energy High Resolution (LEHR) collimator. First, a whole-body scan was acquired to determine lung shunting (140 ± 14 keV, 18 cm/min, 256 pixels wide). Then, a liver-centered SPECT/CT was conducted to estimate the spatial distribution of the ^99m^Tc-MAA (140 ± 14 keV, 32 projections, 30 s/projection, 360°, 128 × 128 pixels). The iterative reconstruction method commercially available used is Astonish® (3 iterations, 8 subsets). ^99m^Tc-MAA lung shunt fraction did not exceed 30 Gy in a single treatment or 50 Gy in case of multiple treatments. In case on an unfavorable ^99m^Tc-MAA work-up, the procedure was repeated and a solution was searched for (e.g., more selective placement of the catheter during injection to improve the targeting of the lesion). If ^90^Y-based SIRT could not be performed, the patient was treated according to best medical practice. If the work-up had a favorable outcome, the patients were re-admitted for treatment within 15 days.

^90^Y Bremsstrahlung Emission Computed Tomography (^90^Y BECT/CT) post-treatment images were acquired on the same Philips BrightView XCT gamma-camera. An energy window around 120 keV ± 24 keV was chosen to avoid the lead fluorescence X-rays around 80 keV and more energetic photons, eventually passing through the collimator because Medium Energy General Purpose (MEGP) collimator was used. First, a whole-body scan was acquired to visually confirm the absence of lung shunting (120 keV ± 24 keV, 12 cm/min, 256 pixels wide). Then, a liver-centered BECT/CT was conducted to estimate the spatial distribution of the glass microspheres (120 keV ± 24 keV, 64 projections, 30 s/projection, 360°, 64 × 64 pixels). The same commercially available algorithm as previously described was used to reconstruct the data. Those ^90^Y BECT/CT were available for both groups but only used for dosimetry purposes when ^90^Y PET/CT wasn’t available, namely for 18 patients (19 treatments) in group A for dose–response assessment. Regarding this standard predictive dosimetry group 0,42 to 5,1 GBq of ^90^Y were injected.

^90^Y PET/CT post-treatment images used here for dosimetry purposes were acquired on a digital Philips Vereos PET/CT scanner (20 min per bed position for a total of 40 min or 2 bed positions, 288 × 288 pixels of 2 × 2 mm with a slice thickness of 2 mm). The iterative reconstruction algorithm is an Ordered Subset Expectation Maximization (OSEM, 3 iterations, 17 subsets, with Point Spread Function correction option applied) (Trotta 2022). Those ^90^Y PET/CT were available for 9 patients (10 treatments) in group B for dose–response assessment. For this personalized predictive dosimetry group 0,26 to 9,84 GBq of ^90^Y were injected.

^90^Y BECT/CT or ^90^Y PET/CT were co-registered and the perfused volume determined (with thresholding based on a maximum intensity percentage of 1%). Personalized dosimetry was performed using Simplicit^90^Y®. MRI or CECT were used to do the segmentation of the liver, the tumor, and the non-tumoral liver (manually on MRI, automatically on CECT with corrections when needed). Lung shunt fraction was evaluated on a Bremsstrahlung Emission Whole Body scan as described above.

In order to compare the received activity according to standard predictive dosimetry and the activity that would have been recommended by the Simplicit^90^Y® software, only patients in *group A* for which we disposed of imaging at 3 months after treatment was available, were included. For all of them, CE-MRI, or CECT, ^99m^Tc-MAA SPECT/CT and ^90^Y BECT/CT have been taken into account.

### Study endpoints

The study had *co-primary endpoints*, the comparison of the objective response rate (ORR evaluated by modified Response Evaluation Criteria in Solid Tumors [mRECIST]) (Lencioni and Llovet 2010), defined by the proportion of treated nodules that presented complete or partial response, as well as the best overall response (BOR) defined as the best recorded response per patient from the start of the study treatment until disease progression between the two groups of patients (i.e., group A including patients treated by ^90^Y with an activity based on a mono-compartmental dosimetry planning and group B using personalized (multi-compartmental) predictive dosimetry using a dedicated software).

*The secondary endpoints* were: (1) the comparison of the two treatment groups in terms of progression free survival (PFS), defined as the time from treatment to the first observation of progressive disease or death, and overall survival (OS), defined as the time from treatment to death of any cause; (2) safety and toxicity profiles in the two groups evaluated according to the Common terminology Criteria of Adverse Events Version 5.0 (CTCAE V5.0) (U.S. Department of Health and Human Services 2017); safety was evaluated clinically and biologically at 1 and 3 months and radiological adverse events (AEs) were recorded at 3 months after treatment; (3) for group A: (i) dose–response link investigation using mRECIST criteria on target lesions at 3 months, (ii) comparison between the activity to be administered by SIRT determined using a personalized dosimetry software with multicompartment MIRD technique (Simplicit^90^Y®) to the activity administered determined by the classical non-compartmental dosimetry planning, using a target volume based on the ^99m^Tc-MAA SPECT only (total perfused volume, for patients treated before the acquisition of Simplicit^90^Y®); (4) for group B, dose–response link investigation using mRECIST (target) criteria at 3 months.

### Statistical analysis

For *group A*, radiological tumor response, evaluated following the mRECIST target criteria at 3 months, was correlated with the perfused tumor dose and the perfused fraction of the total tumor volume, determined using ^90^Y BECT/CT and Simplicit^90^Y®. We also calculated the activity needed to reach 205 Gy in the perfused tumor, using ^99m^Tc-MAA SPECT/CT and Simplicit^90^Y®. The activity to be administered based on this minimal tumor absorbed dose criteria was compared to the activity actually administered. The relative differences were plotted, and correlated these differences with the radiological tumor response.

In *group B*, the radiological tumor response, evaluated following the mRECIST target criteria at 3 months, was correlated with the perfused tumor dose and the perfused fraction of the total tumor volume, determined using ^90^Y PET/CT and Simplicit^90^Y®.

Statistical analysis was performed using Stata/IC version 15.1. Descriptive statistics are reported as means and standard deviations for normally-distributed continuous variables or medians and ranges for asymmetrical distributions, and percentages for categorical variables. Means were compared between the two groups with Student’s *t*-test and asymmetrical distributions were compared with Mann–Whitney–Wilcoxon test. Frequencies were compared between the two groups with Fisher's exact test or Pearson's Chi-squared test, depending on the expected numbers. The number of successful attempts of treatment were compared with a generalized estimating equation (as some attempts were performed on the same patient). PFS and OS were analyzed using the Kaplan–Meier method and compared with the logrank test.

## Results

### Demographics and baseline characteristics

Between February 2016 and December 2020, 66 patients underwent hepatic angiography and ^99m^Tc-MAA scintigraphy in order to evaluate SIRT feasibility. Twenty-nine patients underwent work-up with standard dosimetry (group A) and 37 using personalized dosimetry (group B). Of the 32 evaluations in group A, 9 work-ups were unfavorable and the patients received treatment according to standard guidelines. Six patients had poor targeting (activity spreading outside the goal site) and 3 presented digestive extrahepatic uptake. Twenty-two patients received treatment (75.86%), with one patient undergoing two treatments.

In group B, a total of 47 work-ups were performed, with several patients undergoing more than one evaluation. Thirty failures were registered, mainly due to poor targeting. Only 16 patients received treatment (43.24%), with also one patient undergoing two radioembolizations on the same tumor.

**Fig. 1:**
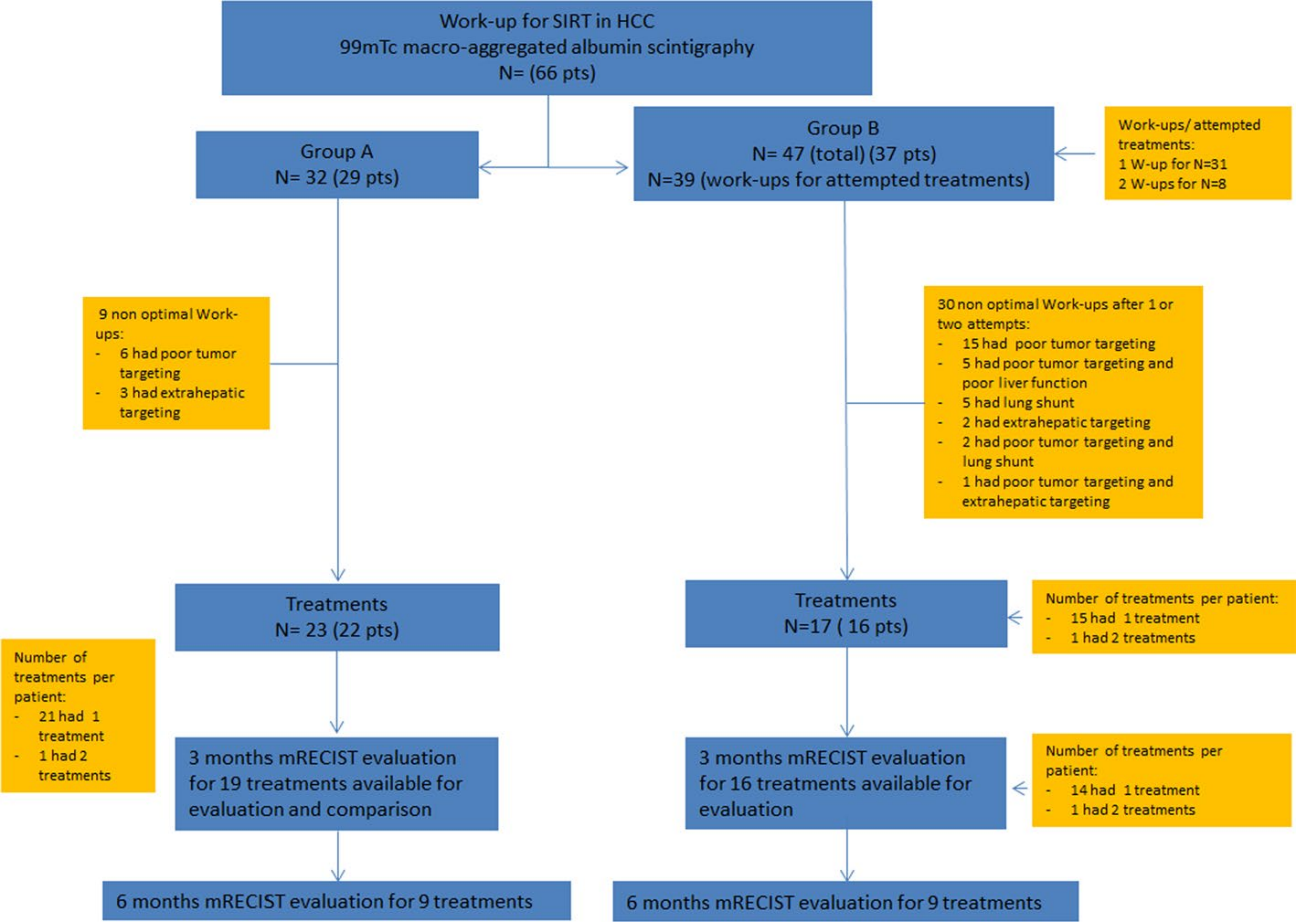
Flowchart of the study (Group A—patients that received SIRT after standard-based-dosimetry simulation; Group B—patients that received SIRT after personalized-based-dosimetry simulation)

The mains reasons for work-up failure are noted in the Flowchart (Fig. 1).

Baseline characteristics of patients in groups A and B receiving SIRT are listed in Table 1. Demographics, tumor burden and liver function were similar for the two groups.

**Table 1:** Baseline characteristics

Baseline characteristics	Group A *N* = 22	Group B *N* = 16 pts	*p*-value
Age (years old) (Mean ± SD)	72.00 ± 9.07	70.25 ± 7.28	0.53
Number patients that received treatment (*n*, %)	22/29 (75.86%)	16/37 (43.24%)	0.008
Gender (male) (*n*, %)	16/22 (72.73%)	14/16 (87.50%)	0.43
Number of successful attempts of treatment (*n*, %)	23/32 (71.88%)	17/39 (43.59%)	0.02
Cirrhosis (*n*, %)	20/22 (90.91%)	12/16 (75%)	0.22
Cirrhosis type (*n*, %)			0.77
Alcohol	10/20 (50%)	6/12 (50%)	1.00
HBV	1/20 (5%)	1/12 (8.33%)	1.00
HCV	3/20 (15%)	3/12 (25%)	0.65
Other (combining factors)	6/20 (30%)	2/12 (16.67%)	0.68
Child–Pugh (*n*, %)			1.00
A	16/18 (88.89%)	11/12 (91.67%)	
B	2/18 (11.11%)	1/12 (8.33%)	
ECOG score (*n*, %)			1.00
0	17/22 (77.27%)	12/16 (75%)	
1	5/22 (22.73%)	4/16 (25%)	
Previous treatment (*n*, %)			0.049
No previous treatment	11/22 (50%)	13/16 (81.25%)	
At least one previous treatment	11/22 (50%)	3/16 (18.75%)	
Previous treatment type (*n*, %)			0.47
SIRT/TACE	5/11 (45.45%)	2/3 (66.67%)	
Systemic	1/11 (9.09%)	1/3 (33.33%)	
Surgery	1/11 (9.09%)	0/3 (0%)	
Multiples combination treatments	4/11 (36.33%)	0/3 (0%)	
HCC-tumor load (*n*, %)			
Infiltrative	2/22 (9.09%)	2/16 (12.50%)	1.00
Satellite	9/22 (40.91%)	5/16 (31.25%)	0.54
Ascites	1/22 (4.55%)	1/16 (6.25%)	1.00
Portal vein invasion	6/22 (27.27%)	4/16 (25.00%)	1.00
Portal hypertension	11/22 (50.00%)	4/16 (25.00%)	0.12
Bile duct dilation	4/22 (18.18%)	4/16 (25.00%)	0.70
Extrahepatic spread	0/22 (0%)	2/16 (12.50%)	0.17
Number of nodules (Median [min–max] HCC)	1 [1–10]	1 [1–3]	0.054
Diameter of biggest nodule (mm) (Median [min–max])	52 [17–163]	65 [13–160]	0.71
AFP Score (*n*, %)			0.74
< 100	14/22 (63.64%)	11/16 (68.75%)	
≥ 100	8/22 (36.36%)	5/16 (31.25%)	
BCLC (*n*, %)			1.00
A	2/22 (9.09%)	2/16 (12.50%)	
B	14/22 (53.64%)	10/16 (62.50%)	
C	6/22 (27.27%)	4/16 (25.00%)	
Treatment characteristics			0.26
Lobar	15/22 (68.18%)	8/16 (50.00%)	
Selective	7/22 (31.82%)	8/16 (50.00%)	

Three out of the 16 patients (18.75%) in group B and 11 out of the 22 patients (50%) patients in group A had a previous treatment (not necessarily on the same lesion).

### Objective response rate (ORR) and best overall response rate (BOR)

No significant statistical difference was found in terms of ORR between the two groups at 3 months evaluation. In group A, ORR of treated nodules was of 68.42% (complete response [CR] of 10.53% and partial response [PR] of 57.89%) while in group B ORR was 87.5% (CR of 31.25% and PR of 56.25%, *p* = 0.24). No response (stable disease [SD]) or progression (progressive disease [PD]) was observed in 31.58% in group A and 12.5% in group B. Nevertheless, 1 patient that presented partial response was shown to have hepatic progression in a non-targeted area; therefore it was classified as PD. The same situation was showed at 6 months evaluation (Table 2A).

**Table 2:** Overall response rate on target areas (A) and best overall response (B – detailed response and C – CR + PR and SD + PD) for the standard dosimetry group at 3 and 6 months, according to mRECIST

(A) mRECIST ORR for HCC treated nodules				
	3 months		6 months	
	Group A *N* = 19	Group B *N* = 16	Group A *N* = 10	Group B *N* = 9
Response (*n*, %)				
CR	2/19 (10.53%)	5/16 (31.25%)	1/10 (10%)	3/9 (33.33%)
PR	11/19 (57.89%)	9/16 (56.25%)	4/10 (40%)	5/9 (55.56%)
SD	3/19 (15.79%)	1/16 (6.25%)	1/10 (10%)	0/9 (0%)
PD	3/19 (15.79%)	1/16 (6.25%)	4/10 (40%)	1/9 (11.11%)

mRECIST ORR for HCC treated nodules			
	Group A *N* = 19	Group B *N* = 16	*p*-value
*Response 3 months: n (%)*			0.24
CR + PR	13 (68.4%)	14 (87.5%)	
SD + PD	6 (31.6%)	2 (12.5%)	
*DCR (CR* + *PR* + *SD) 3 months: n (%)*	16 (84.2%)	15 (93.8%)	0.61

	Group A *N* = 10	Group B *N* = 9	*p*-value
Response 6 months: *n* (%)			0.14
CR + PR	5 (50.0%)	8 (88.9%)	
SD + PD	5 (50.0%)	1 (11.1%)	

(B) BOR according to mRECIST overall				
	3 months		6 months	
	Group A *N* = 18	Group B *N* = 15	Group A *N* = 9	Group B *N* = 9
Response (*n*, %)				
CR	1/18 (5.56%)	5/15 (33.33%)	1/9 (11.11%)	3/9 (33.33%)
PR	5/18 (27.78%)	7/15 (46.67%)	1/9 (11.11%)	4/9 (44.44%)
SD	5/18 (27.78%)	1/15 (6.67%)	1/9 (11.11%)	0/9 (0%)
PD	7/18 (38.89%)	2/15 (13.33%)	6/9 (66.67%)	2/9 (22.22%)

BOR according to mRECIST/overall			
	Group A *N* = 18	Group B *N* = 15	*p*-value
Response 3 months: *n* (%)			0.007
CR + PR	6 (33.3%)	12 (80.0%)	
SD + PD	12 (66.7%)	3 (20.0%)	

	Group A *N* = 9	Group B *N* = 9	*p*-value
Response 6 months: *n* (%)			0.06
CR + PR	2 (22.2%)	7 (77.8%)	
SD + PD	7 (77.8%)	2 (22.2%)	

*CR* complete response, *PR* partial response, *SD* stable disease, *PD* progressive disease according to mRECIST. DCR is defined as the composite of ORR and stable disease between patients in group A and B

When we analyzed the response per patient, in terms of BOR, a significant difference was showed with 80% versus 33.34% (*p* = 0.007) for group B and group A, respectively. This difference was equally observed at 6 months evaluation as showed in Table 2B. Nevertheless, this difference was not persistent for disease control rate at 3 months with 86.7% response in group B versus 61.1% in group A (*p* = 0.15), nor at 6 months with 77.8% for group B versus 33.3% for group A, despite a clear trend for a better efficacy of personalized treatment.

### Follow-up and survival

The *median time of follow-up* was the same in the two groups, 21 months (range 3–55) for group A and 21 months (range 4–39) for the ones in group B.

**Fig. 2:**
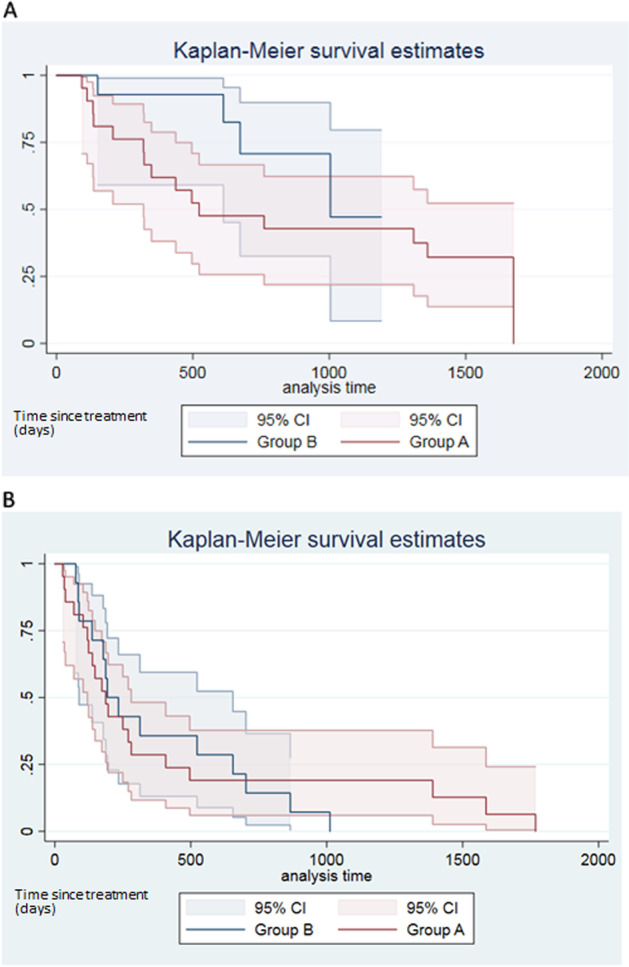
Kaplan–Meier curves for overall survival (OS) (**A**) and progression-free survival (PFS) (**B**). No statistically significant difference was seen between group A and B in OS or PFS

The Kaplan–Meier curves of OS and PFS are depicted in Fig. 2. Median OS in group A was 17.18 months (95% CI: 10.48–not reached) and 32.99 months (95% CI: 20.11–not reached) for group B. When compared, the two groups showed no statistical difference in terms of survival (*p* = 0.17). The same trend was noticed for PFS with a median time to progression in group A of 6.14 months (95% CI: 3.94–9.23) and 6.34 months (95% CI: 2.92–21.52) in group B.

### Safety and complications

In the *safety analysis*, no mortality was registered at 30 days from treatment.

In group A, 6 out of 20 patients (30%) evaluated at 1 months presented grade 1 or 2 clinical AEs (fatigue 33.33%, abdominal pain 50% and nausea/vomiting 33.33%). At three months evaluation, one patient also presented gastrointestinal bleeding due to newly appeared angiomas due to portal hypertension.

**Fig. 3:**
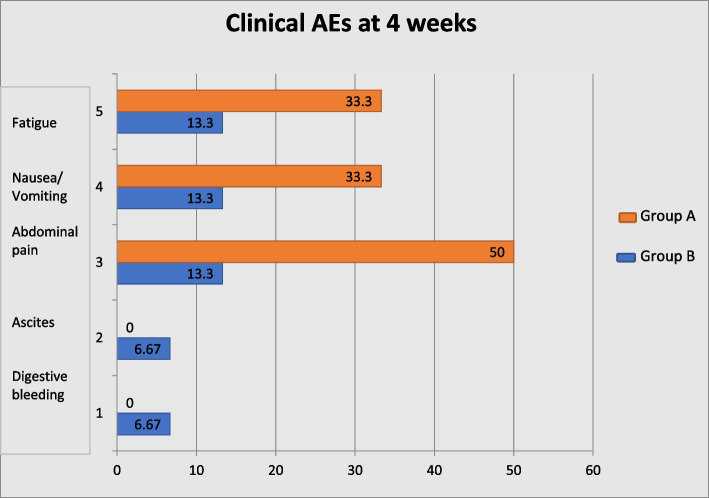
Clinical adverse events (AEs) at 4 weeks

In group B, 7 patients out of 15 (46.67%) that were evaluated at one month presented grade 1 or 2 clinical AEs (nausea 13.33%, fatigue 13.33%, abdominal pain 13.33%, ascites 6.67%, hemorrhagic duodenal ulcer 6.67%). The patient that received two treatments presented a hemorrhagic duodenal ulcer one month after his second treatment. At 3 and 6 months only 3 and 1 patients, respectively, still presented clinical toxicities. The latter had grade 3 ascites that required repeated paracentesis (Fig. 3).

The most common biochemical toxicity at 1 month in group A was hyperbilirubinemia (20%). This persisted at 3 months, with one patient presenting Grade 3 toxicity.

However, the most common biochemical toxicity for group B was decrease albumin at 1 month evaluation (45.45%). No grade 3 or 4 toxicities were shown. No clinical adverse events (AEs) were reported at day 1 after treatment.

In terms of radiological AEs, 5 peritumoral ischemic lesions were shown on CE imaging at three months in group B, with no repercussion on the hepatic function. No biliary injury was noticed. In the patients of group A, 6 ischemic injuries were observed. One patient presented biliary dilation with elevation of blood cholestasis.

### Dose–response link in groups A and B, and retrospective recalculation of activity to be injected in group A using Simplicity^90^Y®

**Fig. 4:**
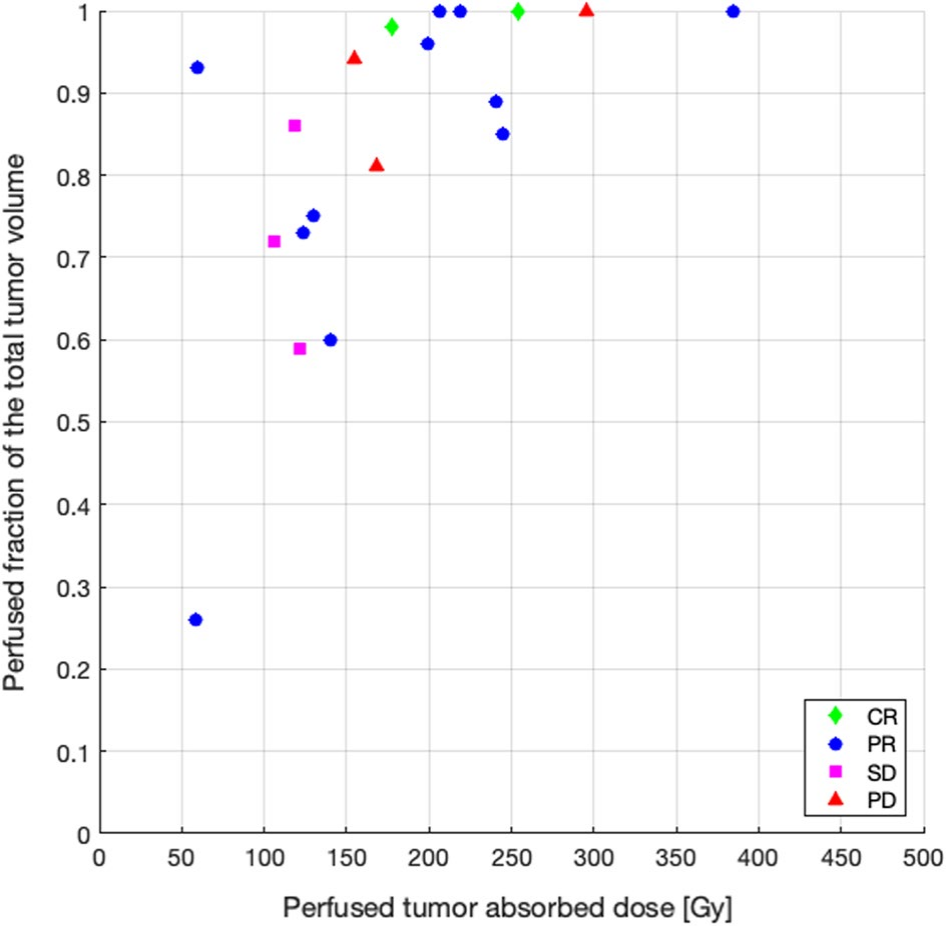
Dot plot of perfused fraction of the total tumor volume according to perfused tumor adsorbed dose and tumor response evaluated following mRECIST (target) criteria at 3 months, in patients from group A with BECT-CT-based dosimetry

**Fig. 5:**
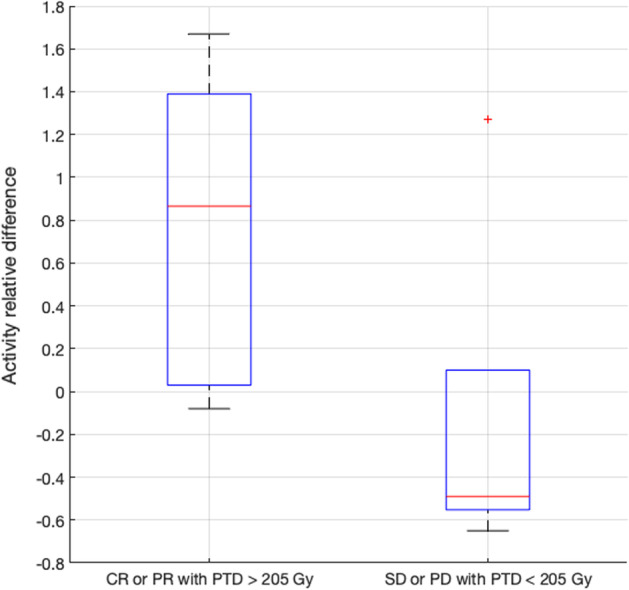
Box plot showing the distribution of activity relative differences (between activity to be administered following personalized dosimetry and activity actually administered following classical standard approach) in 2 subgroups of group A (complete or partial response evaluated at 3 months following mRECIST target criteria with perfused tumor dose above 205 Gy versus stable or progression disease with perfused tumor dose under 205 Gy)

**Fig. 6:**
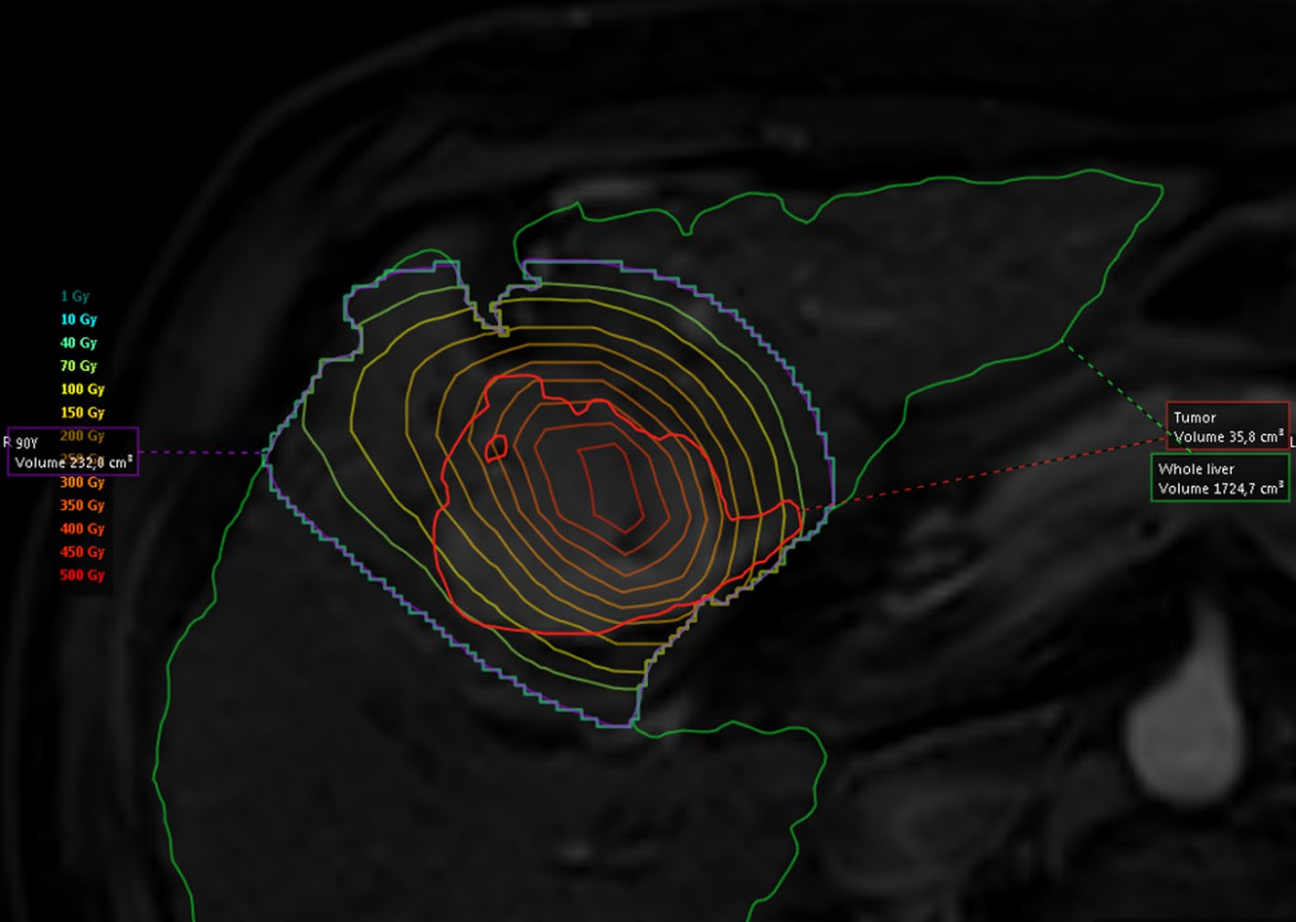
Isodose curves from ^90^Y BECT on MRI images (T2 sequence) in a 61-year-old male patient suffering from an HCC well perfused but non-homogeneously with undertreated parts (under 205 Gy)

Regarding the group A, in order to compare the administered activity according to standard dosimetry and the activity that would have been recommended by the Simplicit^90^Y® software, 18 patients were included, for a total of 19 treatments. For all of them, MRI or CE-CT, ^99m^Tc-MAA SPECT/CT and ^90^Y BECT/CT were available. Eleven among these 19 treatments evaluated by mRECIST (target) criteria at 3 months, induced a PR while 2 induced a CR. In these responders, the ^90^Y BECT/CT-based dosimetry showed 7 patients receiving less than 205 Gy and 6 patients receiving more than 205 Gy in the perfused tumor (188 Gy on average) (Fig. 4). For these latter 6 patients, the comparison between the administered activity and the recommended activity estimated retrospectively using Simplicit^90^Y® showed that 5 patients received an activity higher than recommended (79% mean difference) (Fig. 5), still with a dose to the non-tumoral liver staying below 70 Gy except for 1 patient (44 Gy on average) (Gnesin et al. 2016). Among the 7 responding patients receiving less than 205 Gy in the perfused tumor, 2 of them showed visible areas of necrosis before treatment on the anatomical images used. Among the 6 treatments inducing no response, 3 patients progressed and 3 patients were stable. All of these patients received less than 205 Gy to the perfused tumor, based on a ^90^Y BECT/CT-based dosimetry, except one (Fig. 4). In this latter patient, microspheres were concentrated non-homogenously with a well-defined hot spot and undertreated parts (Fig. 6). The comparison between the administered activity and the recommended activity calculated using Simplicit^90^Y® showed that the other 2 patients who progressed received less activity than recommended (51% mean difference). The results are the same for 2 out of 3 patients who were stable (47% mean difference) (Fig. 5).

**Fig. 7:**
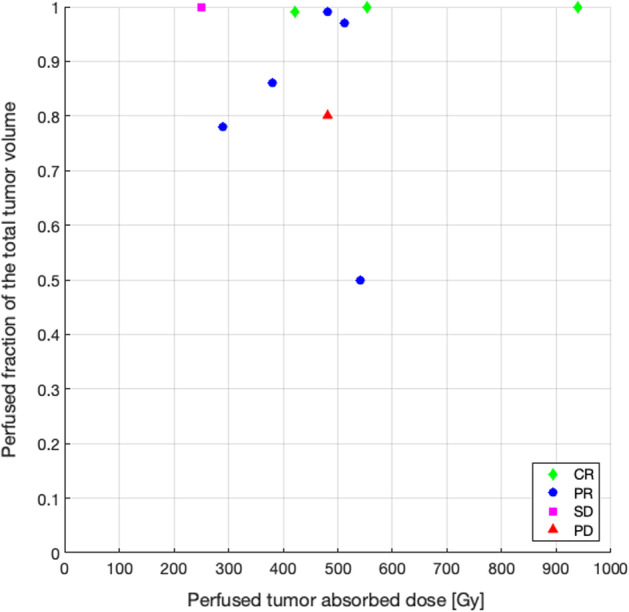
Dot plot of perfused fraction of the total tumor volume according to perfused tumor adsorbed dose and tumor response evaluated following mRECIST (target) criteria at 3 months, in patients from group B with PET-CT-based dosimetry

Nine patients of the group B underwent post-treatment ^90^Y PET/CT (10 treatments). At the 3 months imaging evaluation 8 treatments induced a response (5 PR and 3 CR). Among the 2 treatments inducing no response, 1 patient progressed and 1 patient was stable (Fig. 7). Obviously, all the patients received more than 205 Gy to the perfused tumor. The only patient progressing had a perfused fraction of the total tumor volume of 80%.

## Discussion

Over the last two years SIRT has made a comeback in the treatment planning of HCC. Salem et al. demonstrated in the LEGACY trial that radioembolization provides a durable response for solitary unresectable tumors up to 8 cm in diameter and reestablished its place in the latest BCLC recommendations (Salem et al. 2021; Reig et al. 2022). Furthermore, the DOSISPHERE-01 trial reinforced the importance of a personalized treatment, “tailored” to the tumor and patient. Our retrospective data comparing the objective response rate for treated nodules and best overall response in our population of HCC patients treated using standard predictive dosimetry versus personalized predictive dosimetry confirm the valuable contribution of this new approach.

In our study, there was no significant statistical difference when comparing the two groups in terms of ORR per nodule, despite a trend in favor of personalized dosimetry (87.5% for personalized dosimetry versus 68.4% for standard dosimetry at 3 months, *p* = 0.24). These discordant results are probably due to the small size of our two population groups. By contrast, in the per patient analysis, there was a clear benefit of personalized dosimetry in terms of BOR at 3 months (80% versus 33.3%, *p* = 0.007) and 6 months post-treatment (77.8% versus 22.2%, *p* = 0.06).

The response rate we report is slightly higher than the results published recently in the randomized, multicenter DOSISPHERE-01 study (Garin et al. 2021) using glass microspheres. In this study by Garin et al., a significant statistical difference was shown between objective response rates in favor of personalized predictive dosimetry compared to standard predictive dosimetry (71% versus 36%). However, we note that the diameter of tumors in our study was smaller, with a median of 65 mm, while in the DOSISPHERE-01, at least one nodule had minimum 70 mm. Large tumor size is well known to have a strong negative impact on response and overall survival after locoregional therapies (Kadalayil et al. 2013). This might partially explain the better results in our cohort.

Regarding toxicity, we observed a good safety profile in both groups and more importantly, due to the excellent tolerance and toxicity profile, almost all patients in our study were able to have sequential locoregional or systemic treatment. The preservation of the liver-function is more than essential ever since the development of multiple systemic treatments that improve the overall survival of HCC patients. The final results of the IMBRAVE 150 trial comparing the efficacy of the atezolizumab plus bevacizumab combination versus sorafenib for unresectable HCC showed a median overall survival (OS) of 19.2 vs. 13.4 months; stratified HR 0.66, 95% CI [0.52, 0.85]; *p* = 0.0009) with excellent tolerance (Finn et al. 2020). Moreover, 48% of patients receiving the combination treatment and 52% of those in the Sorafenib arm had already underwent a local therapy before enrollment in the study, suggesting that a locoregional treatment does not prevent sequential treatment.

The DOSISPHERE-01 trial was a breakthrough for SIRT after 2 negative phase III trials (SARAH and SIRveNIB) and one phase II trial (SORAMIC) comparing or evaluating the addition of SIRT to Sorafenib. In terms of overall survival, personalized predictive dosimetry showed an important added value, with 26.6 months for the intention-to-treat population compared to 9.9 months in the SARAH trial and 11.3 months in the SIRveNIB trial. In the SORAMIC trial, the SIRT + sorafenib arm showed a median OS of 12.1 months, with no statistical difference from the Sorafenib arm of 11.4 months. Our study has shown an improvement of OS with personalized dosimetry, even if not statistically significant, 33 months versus 17.2 months for patients treated after standard-dosimetry-based simulation. However, despite the advantage of personalized predictive dosimetry in terms of response, the survival was not impacted. This came as a surprise as a recent study evaluating the relationship between tumor absorbed dose, survival and tumor response in locally advanced inoperable HCC based on the SARAH study population showed that patients who received a higher dose (a threshold of 100 Gy for resin microspheres) had longer survival, 14.1 months [95% CI: 9.6 months, 18.6 months] vs 6.1 months [95% CI: 4.9 months, 6.8 months], respectively; *p* = 0.001) (Hermann et al. 2020). However, this might be also explained by our small population sample included in our study.

With the use of personalized predictive dosimetry, we obtain a better selection of patient that could benefit from SIRT, avoiding inefficient radiotherapies and thus unnecessary dose deposition. However, this leads to an increase of work-up failure. A higher number of patients were not suitable for SIRT after simulation realized with the use of Simplicit^90^Y®, 24.1% for group A versus 56.8% for group B. This point is often overlooked and yet, it is important to point out since patients might undergo an unnecessary hepatic angiography that represents an invasive procedure inducing stress. This drop-out in our study is illustrated in Table 1. In the standard predictive dosimetry group 75.86% of patients received treatment, compared to 43.24% in the personalized predictive dosimetry group, despite more than one work-up attempts. The principal explanation was shown to be poor tumor targeting in 50% of cases, and thus a suboptimal dosimetry (Garin et al. 2017; Gnesin et al. 2016), resulting from the more precise multi-compartmental dosimetry approach available with Simplicit^90^Y®. Our results are higher than those already published in the literature (Garin et al. 2021), probably due to the learning curve (i.e. 1 year lapse between the beginning of SIRT in our institution and the use of personalized treatment), and more strict criteria due to the controversial place of SIRT at the time of the study. We already know that prediction of ^90^Y-microspheres distribution in tumor and non-tumor thanks to the technetium-labeled albumin macroaggregate (^99m^Tc -MAA) pretreatment imaging improves TARE efficacy (Ho et al. 1997a; Ho et al. 1997b). However, more recent data challenged its value to predict the distribution of the Y^90^ in the liver (Wondergem et al. 2013; Ulrich et al. 2013; Ilhan 2015). Newly developed ^166^Holmium microspheres emit not only beta radiation that induces tumor necrosis, but also gamma radiation, which allows for SPECT imaging and the assessment of the radiation absorbed dose delivered in both the tumor and non-tumoral liver. Moreover, holmium is a highly paramagnetic metal, and as such may be visualized by MRI (Nijsen et al. 2001; Nijsen et al. 1999). Another advantage of holmium is the possibility of using a scout dose of ^166^Ho-microspheres for treatment planning instead of ^99m^Tc-MAA. This might improve treatment planning according to a recent publication that showed an advantage of using “the scout dose” over ^99m^Tc-MAA with better distribution of the therapy dose (Smits et al. 2020). The characteristics of these new microspheres and the possibility of using the same product for both work-up and treatment could lead to better treatment planning and patient selection and the ^166^Ho-scout may serve as a predictive biomarker for safe and effective treatment. Holmium studies are ongoing and will offer more data regarding these new microspheres and their safety and efficacy.

However, prediction of microsphere distribution is only one of the aspects of improving patient selection and, subsequently, avoid work-up failure. This needs to start even before the first angiography, with better imaging, such as perfusion MRI/CT. Selecting patients presenting favorable tumor perfusion might decrease the number of patients with unfavorable dosimetry at the simulation and thereby would reduce the number of work-up failure (Radiology 2014, Acad radiol 2019). Moreover, in a recent prospective study, perfusion MRI could represent a tool for evaluation and prediction of HCC response to radioembolization (Radiology Imaging Cancer).

In group A, the comparison between the administered activity and the recommended activity calculated using a personalized dosimetry software with multicompartmental (MIRD) approach showed that the patients who progressed received less activity than recommended (Fig. 5) or that this activity was not adequately distributed (Fig. 6). Our results indicate that reaching a minimal absorbed dose criteria increases the efficacy of SIRT. They support the use of a more personalized predictive dosimetry instead of the classical mono-compartmental MIRD dosimetry for treatment planning. This is in line with the notion that a minimal absorbed dose of a minimum 205 Gy to the lesion is required to achieve an optimal response for SIRT with glass microspheres (Garin et al. 2017; Gnesin et al. 2016). It is worth to note that taking into account areas of necrosis in the tumor absorbed dose calculation does not provide an estimate of the dose to the viable tumor, as mentioned by Garin et al.

Dose–response link evaluations, in both groups, clearly showed that a response cannot be reached if the perfused fraction of the total tumor volume is not enough.

Our study has several limitations, the most important being the small number of patients and its retrospective nature. Furthermore, a bias is to be taken into account due to the fact that some patients had already undergone previous treatments on the treated tumors. Moreover, the groups have been treated in two successive periods. Therefore, the benefit of the experience acquired may have influenced our results for the patients in group B. This effect is mitigated by the fact that the most sensitive part of the protocol is subjected to a learning curve, more precisely the angiographic procedure has been performed by experienced radiologists (between 5–10 years’ experience of hepatic angiography practice).

## Conclusion

Our real-life data aligns with latest findings and advances in radioembolization for HCC. Personalized dosimetry allows a better selection of HCC patients who can benefit from SIRT, and consequently, improves the effectiveness of this treatment.

## Data Availability

All data generated or analyzed during this study are included in this published article.

## References

[CR1] Chow PKH (2018). SIRveNIB: selective internal radiation therapy versus sorafenib in Asia-Pacific patients with hepatocellular carcinoma. J Clin Oncol.

[CR2] Dezarn WA (2011). Recommendations of the American Association of Physicists in Medicine on dosimetry, imaging, and quality assurance procedures for ^90^Y microsphere brachytherapy in the treatment of hepatic malignancies. Med Phys.

[CR3] Finn RS (2020). Atezolizumab plus bevacizumab in unresectable hepatocellular carcinoma. N Engl J Med.

[CR4] Garin E, Rolland Y, Laffont S, Edeline J (2016). Clinical impact of ^99m^Tc-MAA SPECT/CT-based dosimetry in the radioembolization of liver malignancies with ^90^Y-loaded microspheres. Eur J Nucl Med Mol Imaging.

[CR5] Garin E (2017). High impact of macroaggregated albumin-based tumour dose on response and overall survival in hepatocellular carcinoma patients treated with ^90^Y-loaded glass microsphere radioembolization. Liver Int.

[CR6] Garin E (2021). Personalised versus standard dosimetry approach of selective internal radiation therapy in patients with locally advanced hepatocellular carcinoma (DOSISPHERE-01): a randomised, multicentre, open-label phase 2 trial. Lancet Gastroenterol Hepatol.

[CR7] Gnesin S (2016). Partition model-based ^99m^Tc-MAA SPECT/CT predictive dosimetry compared with ^90^Y TOF PET/CT posttreatment dosimetry in radioembolization of hepatocellular carcinoma: A quantitative agreement comparison. J Nucl Med.

[CR8] Grosser OS (2015). Intrahepatic activity distribution in radioembolization with yttrium-90-labeled resin microspheres using the body surface area method—a less than perfect model. J Vasc Interv Radiol.

[CR9] Hermann AL (2020). Relationship of tumor radiation–absorbed dose to survival and response in hepatocellular carcinoma treated with transarterial radioembolization with ^90^Y in the SARAH study. Radiology.

[CR10] Ho S (1997). Tumour-to-normal uptake ratio of ^90^Y microspheres in hepatic cancer assessed with 99Tcm macroaggregated albumin. Br J Radiol.

[CR11] Ho S (1997). Clinical evaluation of the partition model for estimating radiation doses from yttrium-90 microspheres in the treatment of hepatic cancer. Eur J Nucl Med.

[CR12] Ilhan H (2015). Predictive value of ^99m^Tc-labelled MAA scintigraphy for ^90^Y-microspheres distribution in radioembolization treatment with resin microspheres in primary and secondary hepatic tumors. J Nucl Med.

[CR13] Kadalayil L (2013). A simple prognostic scoring system for patients receiving transarterial embolisation for hepatocellular cancer. Ann Oncol.

[CR14] Lencioni R, Llovet JM (2010). Modified recist (mRECIST) assessment for hepatocellular carcinoma. Seminars in Liver Disease Preprint at.

[CR15] Levillain H (2021). International recommendations for personalised selective internal radiation therapy of primary and metastatic liver diseases with yttrium-90 resin microspheres. Eur J Nucl Med Mol Imaging.

[CR16] Nijsen JFW (1999). Holmium-166 poly lactic acid microspheres applicable for intra-arterial radionuclide therapy of hepatic malignancies: Effects of preparation and neutron activation techniques. Eur J Nucl Med.

[CR17] Nijsen JFW (2001). Characterization of poly(L-lactic acid) microspheres loaded with holmium acetylacetonate. Biomaterials.

[CR18] Reig M (2021). BCLC strategy for prognosis prediction and treatment recommendation Barcelona Clinic Liver Cancer (BCLC) staging system. The 2022 update. J Hepatol.

[CR19] Ricke J (2018). The impact of combining Selective Internal Radiation Therapy (SIRT) with sorafenib on overall survival in patients with advanced hepatocellular carcinoma: The SORAMIC trial palliative cohort. Ann Oncol.

[CR20] Salem R (2019). Clinical and dosimetric considerations for Y90: recommendations from an international multidisciplinary working group. Eur J Nucl Med Mol Imaging.

[CR21] Salem R (2021). Yttrium-90 radioembolization for the treatment of solitary, unresectable HCC: the LEGACY Study. Hepatology.

[CR22] Smits MLJ (2020). The superior predictive value of 166Ho-scout compared with ^99m^Tc-macroaggregated albumin prior to 166Ho-microspheres radioembolization in patients with liver metastases. Eur J Nucl Med Mol Imaging.

[CR23] Trotta N (2022). Comparison of PMT-based TF64 and SiPM-based Vereos PET/CT systems for ^90^Y imaging and dosimetry optimization: A quantitative study. Med Phys.

[CR24] U.S. DEPARTMENT OF HEALTH AND HUMAN SERVICES (2017) Common terminology criteria for adverse events (CTCAE).v.5.0. Cancer Therapy Evaluation Program (CTEP)

[CR25] Ulrich G (2013). Predictive value of intratumoral ^99m^Tc-macroaggregated albumin uptake in patients with colorectal liver metastases scheduled for radioembolization with ^90^Y-microspheres. J Nucl Med.

[CR26] Vilgrain V (2017). Efficacy and safety of selective internal radiotherapy with yttrium-90 resin microspheres compared with sorafenib in locally advanced and inoperable hepatocellular carcinoma (SARAH): an open-label randomised controlled phase 3 trial. Lancet Oncol.

[CR27] Weber M (2022). EANM procedure guideline for the treatment of liver cancer and liver metastases with intra-arterial radioactive compounds. Eur J Nucl Med Mol Imaging.

[CR28] Wondergem M (2013). ^99m^Tc-macroaggregated albumin poorly predicts the intrahepatic distribution of ^90^Y resin microspheres in hepatic radioembolization. J Nucl Med.

